# Feasibility, timing and outcome of leadless cardiac pacemaker implantation in patients undergoing cardiac implantable electronic device extraction

**DOI:** 10.1007/s00392-024-02516-0

**Published:** 2024-08-12

**Authors:** Daniel Kiblboeck, Hermann Blessberger, Jakob Ebner, Jakob Boetscher, Julian Maier, Christian Reiter, Joerg Kellermair, Clemens Steinwender, Karim Saleh

**Affiliations:** 1 Department of Cardiology, Kepler University Hospital, Medical Faculty, Johannes Kepler University, Krankenhausstr. 9, 4020 Linz, Austria; 2 Department of Internal Medicine II, Paracelsus Medical University, Salzburg, Austria

**Keywords:** Leadless cardiac pacemaker, Device extraction, Infection, Dysfunction, Outcome

## Abstract

**Background:**

Patients requiring extraction of infected or dysfunctional cardiac implantable electronic devices (CIED) have high morbidity and mortality. The Micra™ leadless cardiac pacemaker (LCP) may be beneficial for patients requiring permanent pacemaker therapy after CIED extraction.

**Methods:**

This study aimed to assess the feasibility, timing and outcomes of LCP implantation in patients who underwent CIED extraction due to infection or dysfunction. The local Micra™ LCP registry was reviewed for LCP implantations and CIED extractions.

**Results:**

Micra™ LCP implantation was scheduled for 48 consecutive patients (21 women, 44%) undergoing CIED extraction for infection (*n = *38, 79%) or dysfunction (*n = *10, 21%), and feasible in 47 (98%). Complete CIED removal was feasible in 44 patients (92%) and in 37/38 patients with infected CIED (97%). Overall, 32 LCP (67%) were implanted in a single procedure: 3 (6%) before and 13 (27%) after CIED extraction. LCP were implanted in a single procedure in 24/38 patients (63%) with infected CIED and in 8/10 patients (80%) with dysfunctional CIED. The in-hospital mortality rate was 6% (*n = *3), and the survival rates at 30 days, 90 days and 1 year were 94% (*n = *45/48), 90% (*n = *43/48), and 85% (*n = *41/48), respectively. No recurrent LCP-related mortality or infections occurred during a median follow-up of 15 (interquartile range, 12–41) months.

**Conclusion:**

Two-thirds of LCPs could be implanted in a single procedure with CIED extraction; no recurrent infections were detected. Overall, Micra™ LCP implantation in patients requiring CIED extraction was feasible.

**Graphical Abstract:**

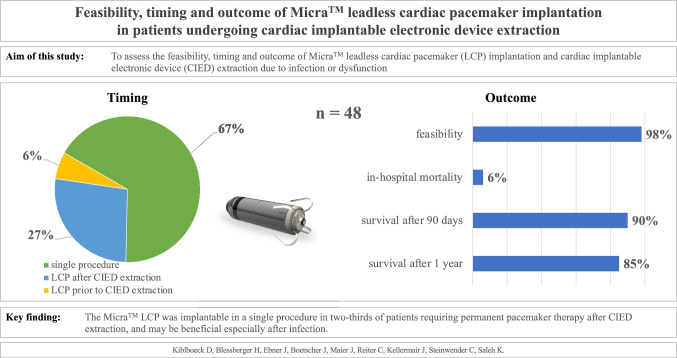

## Introduction

Cardiac implantable electronic devices (CIEDs) have improved the quality of life of patients with arrhythmias and reduced arrhythmia-related mortality in recent decades. However, Kirkfeldt et al. reported device- or lead-related complications, such as hematomas, infections, lead dislocations, and perforations, in 9.5% of patients with cardiac implantable electronic devices [1].These device-related complications may require a CIED revision and/or lead extraction. A nationwide study in the US reported an increasing number of CIED-related infections, with mortality rates of patients undergoing device extraction ranging from 4 to 5.8% [2]. The safety of the Micra™ leadless cardiac pacemaker (LCP) system has been demonstrated in several studies [3–6].The Micra Transcatheter Pacing Study reported an implantation success rate of 99.2%, and pacing capture thresholds remained low and stable in 96.0% of the study population [3]. Duray et al. confirmed the long-term safety of Micra™ LCP with a 96% freedom from major system- or procedure-related complication rates after 12 months [4]. There was an even lower rate of major complications (1.6%) in the Micra post-approval registry in a real-world setting [5]. Only rare cases of infected LCP have been reported [7, 8]. Therefore, LCPs may be a beneficial therapeutic concept in comparison with conventional transvenous pacemakers for patients requiring CIED extraction, especially in patients with infected CIEDs. Recent studies demonstrated the feasibility of this new therapeutic concept [9–12]. However, data on LCP implantation in patients undergoing CIED extraction are limited. This study aimed to investigate the feasibility, timing, and outcomes of CIED extraction and LCP implantation at an experienced, high-volume LCP implantation and CIED extraction center.

## Methods

### Study design and outcomes

The local LCP registry at our institution was analyzed for patients with Micra™ LCP (Medtronic, Minneapolis, MN, USA) implantation undergoing CIED extraction because of infection or dysfunction. The primary objectives of this single-center study were to determine the timing of CIED extraction and LCP implantation and the feasibility of this therapeutic concept. Feasibility was defined as the successful extraction of the CIED and Micra™ LCP implantation. In addition, we analyzed the causes of CIED extraction, microbiological culture results, in-hospital mortality, and causes of death and survival rates after 30 and 90 days and 1 year.

This study was conducted at an experienced high-volume CIED center for LCP implantation and CIED extraction. The study design was approved by the local ethics committee (EC number 1277/2019) and patient informed consent was waived by the ethics committee. The study was conducted in accordance with the Declaration of Helsinki.

### Implantation procedure

All procedures were performed under general anesthesia and standby by a cardiothoracic surgeon and a perfusionist with a primed heart–lung machine in a hybrid operating room. The main operator was an interventional cardiologist, and the cardiothoracic surgeon was present in the operating room during lead extraction.

**Fig. 1:**
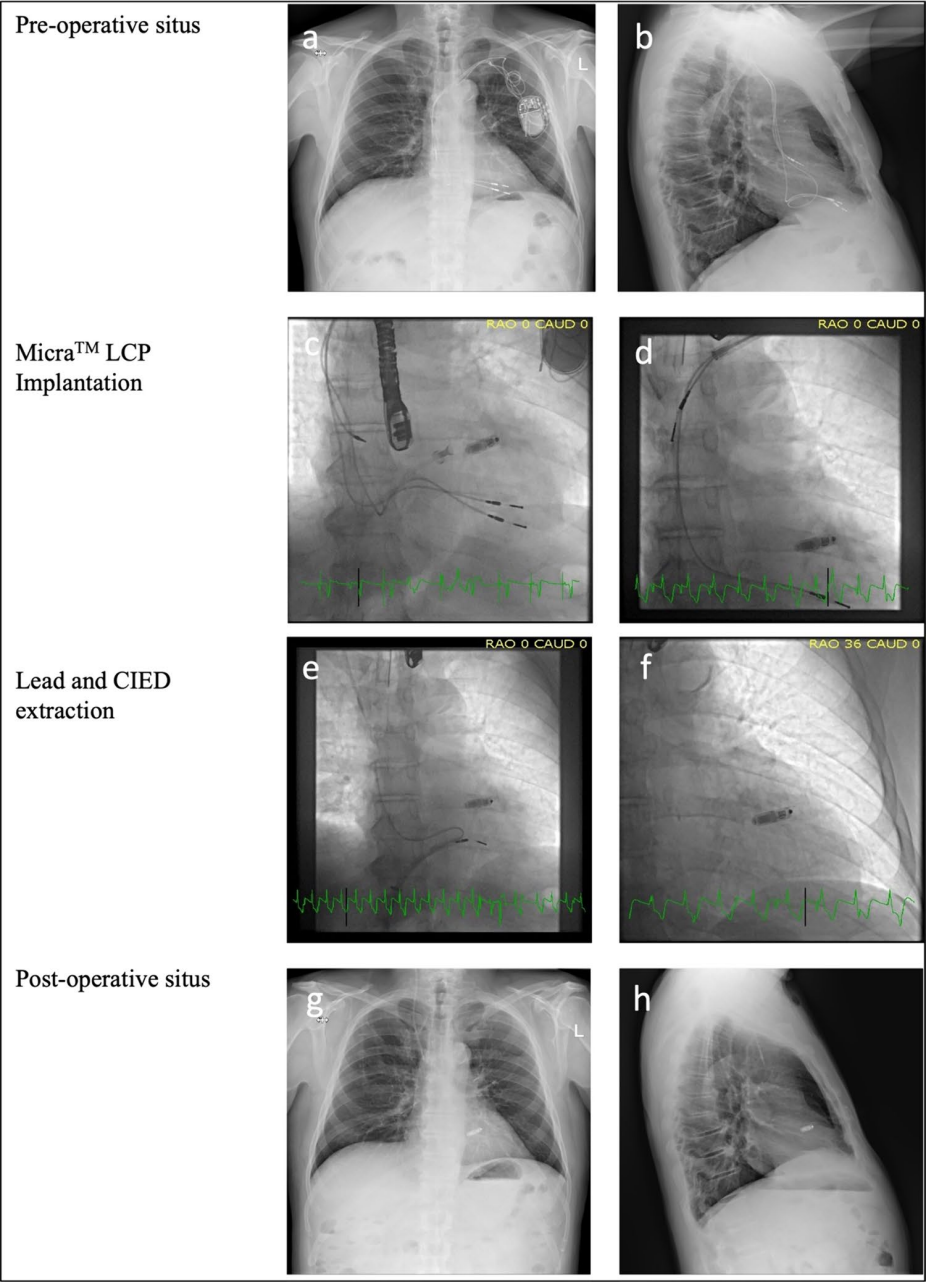
LCP implantation and CIED extraction in a single procedure. The pre-operative situs (**a**, **b**), the Micra™ LCP implantation (**c,** **d**), the lead and CIED extraction procedure (**e**, **f**) and the post-operative situs (**g**, **h**). *CIED* cardiac implantable electronic device, *LCP* leadless cardiac pacemaker

Implantation of the transcatheter Micra™ LCP through the femoral vein, as described elsewhere [13–15], took place in a single procedure (Fig. 1) immediately before or after lead extraction or in a second procedure a few days prior to or after CIED extraction. In the case of LCP implantation prior to extraction, the LCP was placed in the right ventricle at a distance of at least one centimeter from the tip of the lead intended to be extracted. Transvenous lead extraction was performed using standard equipment and technology [16]. Lead locking stylets (Cook Liberator® Beacon® Tip Locking Stylet, Cook Medical, Bloomington, IN, US) as well as mechanical powered sheaths (Evolution® RL, Cook Medical) were used via the subclavian approach. In some cases, a femoral approach was chosen using snare technology with the Cook Needle’s Eye Snare (Cook Medical) or ONE-Snare (Merit Medical Systems, Inc. South Jordan, UT, US) and if necessary, when a superior vena cava tear occurred a Bridge Occlusion Balloon® (Philips, San Diego, CA, US) was deployed through the Micra™ introducer sheath. Pacemaker dependency was defined as a pacing percentage of at least 80% at follow-up 3 months after LCP implantation. Perioperative antibiotic therapy was administered according to the current ESC Guidelines for the management of infective endocarditis with an initial empirical treatment that was de-escalated according to microbiological and antibiogram results [17].

### Statistical analysis

Categorical parameters are described as absolute numbers and percentages. Continuous values are presented as median and interquartile range (IQR). Calculations, including Kaplan–Meier analysis, were performed using the software package Intercooled STATA (release 14.0, StataCorp LP, Texas, USA).

## Results

### Study population

Between August 2015 and September 2022, 48 patients were admitted because of infection or dysfunction for CIED extraction and consecutive Micra™ LCP implantation at our institution and representing the study population. The median age of the study population was 79 years (IQR 71–84 years). Twenty-one patients (*n = *21/48, 44%) were women. Thirty-eight CIEDs (*n = *38/48, 79%) were extracted because of the following infections: pocket infection (*n = *19/38, 50%), lead infection/endocarditis (*n = *10/38, 26%), and pocket perforation with infection (*n = *9/38, 23%). Microbiological cultures of the extracted devices and blood cultures were positive in 22 patients (58%) with infected CIEDs. The most frequent pathogens were Staphylococcus species (Staphylococcus aureus, *n = *10; methicillin-resistant, *n = *1; epidermidis, *n = *6; hominis, *n = *1; hemolyticus, *n = *1), beta-hemolytic Streptococcus (*n = *1), Pseudomonas aeruginosa (*n = *1), Arthrobacter species (*n = *1), and co-infection with Candida albicans (*n = *1). Ten CIEDs (21%) required extraction because of dysfunction, including severe tricuspid regurgitation due to pacemaker leads (*n = *5/10, 50%), lead failure (*n = *4/10, 40%), and chronic pain due to the CIED (*n = *1/10, 10%). The median ejection fraction of the study population was 55% (IQR: 50–60%). The baseline data and further details, including comorbidities, of the study population are presented in Table 1.

**Table 1:** Baseline data of the study population (*n = *48)

Parameter	Median (IQR) or count (%)
Age (years)	79 (71–84)
Sex (female)	21 (44)
Body mass index (kg/m^2^)	25.5 (23.2–29.4)
Arterial hypertension	33 (69)
Diabetes mellitus type 2	12 (25)
Hypercholesterinemia	24 (50)
Coronary artery disease	11 (23)
History of acute myocardial infarction	3 (6)
EF (%)	55 (50–60)
TAPSE (mm)	19 (15–23)
NT-pro-BNP (pg/ml)	1532 (820–2501)
Paroxysmal atrial fibrillation	10 (21)
Persistent atrial fibrillation	5 (10)
Permanent atrial fibrillation	18 (38)
History of stroke	9 (19)
Peripheral artery disease	2 (4)
Chronic obstructive pulmonary disease	4 (8)
Chronic kidney disease stage 1 and 2	25 (52)
Chronic kidney disease stage 3	15 (31)
Chronic kidney disease stage 4	5 (10)
Chronic kidney disease stage 5	3 (6)
Neoplasia	6 (13)
Betablocker	28 (58)
ACE inhibitor/ARB/renin inhibitor/MRA	25 (52)
Novel oral anticoagulants / oral anticoagulants	36 (75)
Antiplatelet therapy	7 (15)

*ACE* angiotensin-converting-enzyme, *ARB* angiotensin receptor blockers, *EF* ejection fraction, *eGFR* estimated glomerular filtration rate, *IQR* interquartile range, *MRA* mineralocorticoid receptor antagonist, *TAPSE* tricuspid annular plane systolic excursion

### CIED removal

Overall, 34 dual-chamber (DDD) pacemakers (71%), 9 single- chamber (VVI) pacemakers (19%), 2 cardiac resynchronization therapy (CRT)-pacemakers (CRT-Ps) (4%), 2 implantable cardioverter defibrillators (ICDs) (4%), and 1 CRT-defibrillator (CRT-D) (2%) were extracted by two experienced interventional cardiologists with standby cardiothoracic surgeons. Complete removal of the CIED, including all leads, was feasible in 44 patients (92%) and 37 patients with infected CIEDs (*n = *37/38, 97%). One left ventricular (LV) Starfix™ lead, one right ventricular (RV) lead, and one right atrial (RA) lead could not be completely extracted, and one lead could only be partially removed, with a remnant in the femoral vein. Pericardial effusion requiring pericardiocentesis occurred in two female (4%), hemodynamically stable pericardial effusion without pericardiocentesis in one female (2%), and local hematoma requiring reoperation in one male and two female patients (6%). No emergency thoracotomy was required for CIED extraction. Overall, a higher rate of complications was observed in women (women: *n = *5/21, 24%; men: *n = *1/27, 4%).

### Micra™ LCP implantation, feasibility and timing

Overall, 32 patients (67%) were scheduled to undergo Micra™ LCP implantation with CIED extraction in a single procedure. Three LCPs (6%) were implanted prior to the CIED extraction procedure (median, 2 days; IQR, 1–2 days) and 13 (27%) after (median, 6 days; IQR, 3–11 days). With respect to infected CIEDs (*n = *38), 24 LCPs (63%) were implanted in a single procedure, 2 LCPs (5%) before and 12 LCPs (32%) after the extraction procedure. Eight LCPs (80%) implanted because of dysfunctional CIED were implanted in a single procedure: one LCP (10%) prior to and one LCP (10%) after. Thirteen patients (27%) in our study population were pacemaker dependent, 12 (92%) of whom underwent LCP implantation and CIED extraction in a single procedure, and a temporary pacemaker was used in one patient.

Overall, feasibility, defined as successful CIED extraction and LCP implantation, was 98% (*n = *47). One patient experienced cardiac arrest due to severe sepsis immediately after CIED extraction and died before LCP implantation. Thirty-four patients (71%) received the Micra™ VVI LCP, and 13 patients (27%) received the Micra™ atrioventricular (AV) LCP. Median sensing, pacing threshold, and impedance values at implantation were 8.1 mV (IQR: 7.0–11.5 mV), 0.38 V/0.24 ms (IQR: 0.25–0.60 V/0.24 ms) and 810 Ohm (IQR: 650–930 Ohm), respectively. Sensing, pacing threshold, and impedance values remained stable after 3 months: 13.4 mV (IQR: 9.7–19.0 mV), 0.38 V/0.24 ms (IQR: 0.38/0.24 ms–0.50 V/0.24 ms) and 580 Ohm (IQR: 525 – 680 Ohm), respectively. Procedural data are presented in Table 2.

**Table 2:** Procedural data of LCP implantation and CIED extraction (*n = *48)

Parameter	Median (IQR) or count (%)
Pacing indication	
2nd degree AV-block Mobitz II (intermittent or permanent)	2 (4)
3rd degree AV-block (intermittent or permanent)	18 (38)
Atrial fibrillation with slow conduction	12 (25)
Sick sinus syndrome	15 (31)
Pace and ablate for atrial fibrillation with rapid conduction	1 (2)
Previous CIED operations	
New implanted CIED	27 (56)
Generator change	14 (29)
Lead revision	11 (23)
Generator change and lead revision	5 (10)
Rhythm at LCP implantation	
Atrial fibrillation	29 (60)
Sinus rhythm	19 (40)
Position, deployments, and type of LCP	
Septal	39 (81)
Apical	7 (15)
Deployments	1 (1–1)
Micra VVI LCP	34 (71)
Micra AV LCP	13 (27)
Successful implantation of LCP	47 (98)
LCP parameters at implantation	
Sensing (mV)	8.1 (7.0–11.5)
Pacing threshold (V/0.24 ms)	0.38 (0.25–0.60)
Impedance (Ohm)	810 (650–930)
Baseline stimulation rate (/min)	60 (50–60)
LCP parameters at follow-up after 3 months	
Sensing (mV)	13.4 (9.7–19.0)
Pacing threshold (V/0.24 ms)	0.38 (0.38–0.50)
Impedance (Ohm)	580 (525–680)
Pacing percentage (%)	83 (10–100)
CIED extraction procedure	
VVI	9 (19)
DDD	34 (71)
CRT-P	2 (4)
CRT-D	1 (2)
ICD	2 (4)
Complete removal of CIED including all leads	44 (92)
Timing of LCP implantation and CIED extraction	
Single procedure on the same day	32 (67)
LCP implantation prior to CIED extraction	3 (6)
Days between LCP implantation and CIED extraction	2 (1–2)
LCP implantation after CIED extraction	13 (27)
Days between CIED extraction and LCP implantation	6 (3–11)
Causes for CIED extraction	
Pocket infection	19 (40)
Lead infection/endocarditis	10 (21)
Pocket perforation	9 (19)
Lead failure	4 (8)
Severe tricuspid regurgitation due to pacemaker lead	5 (10)
Chronic pain (e.g., shoulder) due to pacemaker	1 (2)
Microbiological results (blood culture or device swab)	
Staphylococcus aureus	10 (21)
Staphylococcus epidermidis	6 (13)
Methicillin resistant staphylococcus aureus	1 (2)
Staphylococcus hemolyticus	1 (2)
Staphylococcus hominis	1 (2)
Beta-hemolytic streptococcus	1 (2)
Pseudomonas aeruginosa	1 (2)
Arthrobacter	1 (2)
Candida albicans	1 (2)
Complications	
Pericardial effusion requiring pericardiocentesis	2 (4)
Pericardial effusion without requiring pericardiocentesis	1 (2)
Local hematoma requiring reoperation	3 (6)
Emergency thoracotomy	0 (0)

*AV* atrioventricular, *CIED* cardiac implantable electronic device, *CRT-D* cardiac resynchronization therapy-defibrillator, *CRT-P* cardiac resynchronization therapy-pacemaker, *ICD* implantable cardioverter defibrillator, *IQR* interquartile range, *LCP* leadless cardiac pacemaker

The CIED system was downgraded to LCP in five patients (10%; CRT-P, *n = *2; ICD, *n = *2; CRT-D, *n = *1) in our study population. One CRT-P was extracted because of severe tricuspid valve regurgitation due to the pacemaker lead, and another CRT-P because of an infected epicardial lead. Both patients were pacemaker-dependent after AV nodal ablation for atrial fibrillation with rapid conduction, and the LCP was implanted in a single procedure immediately before CIED extraction. One patient with dilated cardiomyopathy was downgraded from ICD to LCP because he had not received shocks since the first ICD implantation 17 years ago. The second patient with an ICD no longer required another device, as he was already 88 years old. Both patients had a pocket infection. One CRT-D could be downgraded after chart review with a primary indication for hypertrophic non-obstructive cardiomyopathy with an ejection fraction of 55%, initial non-sustained ventricular tachycardias, and syncopal pauses with a low estimated risk of sustained ventricular arrhythmias.

### Mortality and survival

**Fig. 2:**
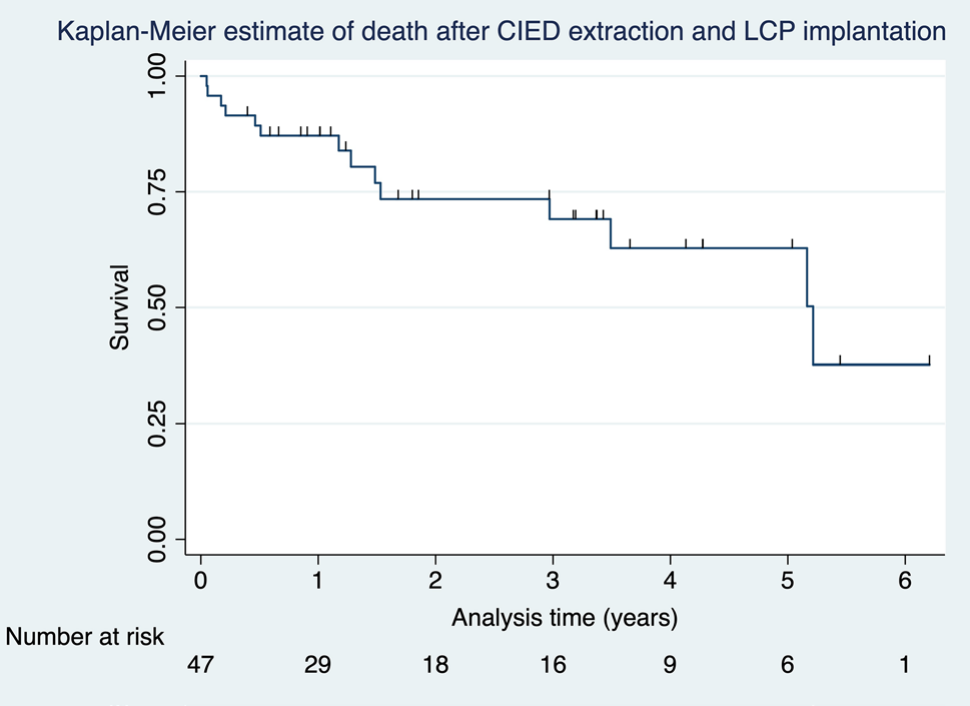
Kaplan–Meier estimates of death after CIED extraction and LCP implantation for all patients (*n = *48). *CIED* cardiac implantable electronic device, *LCP* leadless cardiac pacemaker

The overall in-hospital mortality rate was 6% (*n = *3/48), including 8% (*n = *3/38) of infected CIEDs and 0% (*n = *0/10) of dysfunctional CIEDs. The causes of death during the hospital stay were cardiac arrest due to severe sepsis after CIED extraction and death before successful LCP implantation. Two other patients died of sepsis and a complicated pulmonary abscess after CIED extraction with successful LCP implantation. The survival rate after 30 days was 94% (*n = *45/48). During follow-up, another two patients died of myocardial infarction and bladder cancer with metastases, resulting in a survival rate of 90% (*n = *43/45) after 90 days. Forty-one patients (85%) survived for one year. Kaplan–Meier estimates of death after CIED extraction and LCP implantation for all patients are presented in Fig. 2. Causes of death after hospital discharge were not related to CIED extraction or the LCP implantation procedure during a median follow-up of 15 months (IQR: 12–41 months). These details are presented in Table 3. No recurrent infections were detected at the pacemaker outpatient clinic during follow-up visits. Additionally, fluorodeoxyglucose positron emission tomography/computed tomography (FDG PET/CT), which was performed in 7 of 38 patients (18%) with infected CIED at a median of 3 months (IQR: 1–3 months) after LCP implantation, did not demonstrate LCP hypermetabolism.

**Table 3:** Outcome data

Parameter	Count (%)
In-hospital mortality	3 (6)
Survival status (*n = *48)	
Alive at 30 days	45 (94)
Alive at 90 days	43 (90)
Alive at 1 year	41 (85)
Deceased patients with age at LCP implantation and causes of death (causes for CIED extraction, removal status) (*n = *15)	Time to death (days)
ID211, female, 83 years, septic shock with cardiac arrest after CIED extraction (lead endocarditis with Pseudomonas aeruginosa, complete removal)	0
ID262, male, 87 years, pulmonary abscess (lead endocarditis with negative microbiological results, complete removal)	18
ID353, female, 67 years, sepsis (lead endocarditis with Staph. aureus, complete removal)	21
ID322, male, 81 years, myocardial infarction (pocket infection with negative microbiological results, complete removal)	63
ID296, male, 73 years, bladder cancer with metastases (pocket infection with negative microbiological results, complete removal)	77
ID325, male, 88 years, congestive heart failure (pocket infection with Staph. aureus, complete removal)	169
ID383, female, 87 years, congestive heart failure (pocket perforation with Staph. epidermidis, complete removal)	186
ID173, female, 60 years, sepsis with multiple ulcera (lead endocarditis with negative microbiological results, complete removal)	429
ID130, male, 81 years, dissection of arteria basilaris and aspiration pneumonia (pocket perforation with negative microbiological results, complete removal)	467
ID308, female, 86 years, marasmus senilis (pocket perforation with negative microbiological results, complete removal)	542
ID68, female, 84 years, congestive heart failure with renal failure (pocket infection with Staph. aureus, complete removal)	559
ID136, male, 82 years, pulmonary edema and severe tricuspid regurgitation (severe tricuspid regurgitation due to pacemaker lead, complete removal)	1085
ID142, male, 79 years, congestive heart failure (lead failure, incomplete removal of RV lead)	1471
ID59, male, 84 years, diabetes mellitus (pocket infection with Staph. aureus, complete removal)	1886
ID69, female, 71 years, ileus (pocket infection with negative microbiological results, complete removal)	1905

*CIED* cardiac implantable electronic device, *LCP* leadless cardiac pacemaker

## Discussion

This observational registry study demonstrated that Micra™ LCP implantation in patients undergoing CIED extraction for infection or dysfunction was feasible in 98% of cases and could be performed in two-thirds of the study participants in a single procedure. The in-hospital mortality was 6%, and survival rates demonstrated a good outcome, with survival rates of 94, 90, and 85% after 30 days, 90 days, and 1 year, respectively (Table 3). The causes of death after hospital discharge were not related to LCP implantation or CIED extraction procedures. No recurrent LCP infections occurred during follow-up (median, 15 months; IQR: 12–41 months).

### Feasibility and timing

CIED-related complications, particularly infections and dysfunction, may require reoperation with CIED extraction. These procedures are high-risk interventions usually performed by experienced interventional cardiologists with standby cardiothoracic surgeons. According to a nationwide study in the United States, patients undergoing CIED extraction due to infections have high mortality rates ranging from 4 to 5.8% [2]. In a study conducted in Belgium, systemic CIED infection was the only significant predictor of 30-day mortality [18]. In our study population, 38 patients (*n = *38/48; 79%) required CIED extraction because of infections, particularly pocket infection, lead infection/endocarditis, and pocket perforation.

Re-infection of LCPs is rare, and only a few cases have been reported in the literature [7, 8]. Postmortem analysis of LCPs demonstrated a fibrous capsule around an implanted LCP in the right ventricle, which may protect the surface against device infections in patients with bacteremia [19, 20]. Several other potential mechanisms of LCPs bacterial resistance, such as absent subcutaneous pocket, smaller surface area of hardware in bloodstream with turbulent and increased velocity fluid hemodynamics and none to minimal handling of the device compared to conventional transvenous pacemakers, were discussed in a review by El-Chami et al. [21]. Therefore, LCPs appear to be a promising new treatment option for patients who require permanent pacemaker therapy after CIED extraction for infection. However, a prerequisite according to the EHRA expert consensus on implantation for CIEDs is that patients should be free of active infection and afebrile for >24 h [22]. Our study group previously reported a case series of six patients with severe CIED infections requiring extraction who underwent LCP implantation [10]. No recurrent infections of the LCP were detected at follow-up with FDG PET/CT after a median of 3 months. El-Chami et al. reported on 105 patients in the Micra post-approval registry with LCP implantation at a median of 6 days after CIED extraction and 37% on the same day of LCP implantation [11]. Another study by Bicong et al. described 39 patients with CIED infection and successful LCP implantation of nine patients in a single procedure and 30 patients after their extraction [23]. 184 of the 1179 patients, who were enrolled in the International Leadless Pacemaker Registry, underwent LCP implantation after a median of 12 (IQR 9–15) days after the CIED extraction procedure [24]. In contrast, our study demonstrated an even higher proportion of patients who underwent CIED extraction and LCP implantation as a single procedure (overall, *n = *32/48; 67%). The main reason for LCP implantation in a second procedure after CIED extraction was severe systemic infections/sepsis requiring antibiotic treatment and intensive care support. Therefore, correct timing for CIED extraction and LCP implantation is crucial for the clinical outcomes and survival of these patients.

Ten patients (21%) required CIED extraction because of dysfunctions such as severe tricuspid regurgitation due to pacemaker leads, lead failure, and chronic pain (e.g., shoulder) due to the device. An LCP could be implanted in eight of these patients in a single procedure (80%). One patient with severe tricuspid regurgitation due to pacemaker lead received the LCP 2 days prior to CIED extraction and one patient 2 days after surgical repair of severe tricuspid regurgitation and CIED extraction.

Most patients received a Micra™ VVI LCP (*n = *34/48; 71%), and 13 patients (27%) received a Micra™ AV LCP for AV synchronous pacing after the DDD pacemaker explant. Therefore, the Micra™ LCP may be a therapeutic approach for patients with 2-chamber DDD pacemakers. However, the major limitation of this approach might be the AV synchrony of 70–89% of VDD pacing in younger patients with complete AV block. As mentioned in detail in the results, LCPs may also be a therapeutic option in patients with a CIED system downgraded from CRT-D/P and ICD when the estimated risk of sustained ventricular arrhythmias changes, especially in older patients with reduced life expectancy. However, it is important to consider, that LCP implantation after CIED extraction might pose patients at risk for undertreatment. Therefore, outweighing risks and benefits of LCP, such as pacemaker induced cardiomyopathy versus a lower risk of reinfection, and conventional pacemaker therapy, such as reinfection or access complications after CIED extraction versus CRT, is crucial to achieve a shared decision making with the patient for an individualized and optimal treatment strategy.

### Outcome and complications

The outcome data demonstrated overall in-hospital mortality rates of 6% (*n = *3/48) and 8% (*n = *3/38) in patients with infected CIEDs. The survival rates at 30 days, 90 days and 1 year were 94% (*n = *45/48), 90% (*n = *43/48), and 85% (*n = *41/48), respectively. The causes of death after hospital discharge were not related to CIED extraction or LCP implantation (Table 3). These results are comparable to those of other studies. Deckx et al. reported a 30-day mortality rate of 3.4% (*n = *6) in a larger cohort of 176 patients who underwent CIED extraction for infection or dysfunction [18]. Remarkably, the patients with systemic CIED infections had a higher 30-day mortality rate (19%). These findings were confirmed by our study results with higher in-hospital mortality rates in patients with infected CIEDs, including pocket infections, pocket perforations, and lead infections (*n = *3/38, 8%). Recently, Beccarino et al. reported comparable results in 86 patients undergoing transvenous lead extraction for active infections with concomitant LCP implantation [25]. The mortality rate was 29% after a median follow-up of 163 days. However, 88% of the deaths were unrelated to the initial infection. Their findings and ours demonstrate that the outcome after CIED extraction and LCP implantation is predominately determined by additional factors such as age and comorbidities. In addition, as demonstrated by Segreti et al., female patients undergoing transvenous lead extraction were at higher risk for major complications and procedural mortality [26]. Precautions, such as sex-specific protocols and techniques, may improve the outcome for women.

### Limitations

Our study had several limitations. First, this was a monocenter registry study and not a prospective randomized trial comparing a new single procedure (concomitant Micra™ LCP implantation and CIED extraction) vs. two standard procedures (Micra™ LCP implantation before or after CIED extraction). The timing for CIED extraction and Micra™ LCP implantation was decided for each patient individually based on cause (infection or dysfunction), type of CIED (single-chamber, dual-chamber pacemakers, CRT-D/P or ICD) and co-morbidities, as this may be crucial for the outcome. The presented study results reflect the experiences of this new therapeutic concept for patients requiring permanent pacemaker therapy after CIED extraction for infection or dysfunction in a highly experienced center for Micra™ LCP implantation and CIED extraction.

Second, the Micra™ LCP VVI and AV are suitable for patients requiring VVI and DDD pacemakers. Complex CIEDs (CRT-P/D and ICD) represent only 10% of the study population leading to a selection bias of simpler CIEDs with less severe comorbidities compared to patients requiring CRT or ICD for heart failure and / or ventricular arrhythmias. Although patients with CRTs can be downgraded with a LCP, a new LCP for LV pacing is needed to optimize outcomes in this patient population. As an alternative for patients requiring ICD therapy, a subcutaneous ICD may be used in combination with an LCP for antitachycardia pacing [27].

Third, the study results are limited to Micra™ LCP (Medtronic, Minneapolis, MN, USA). The LCP implantation and CIED extraction were performed by a highly experienced multidisciplinary team of interventional cardiologists, heart surgeons stand-by in the room, perfusionists with a primed heart–lung machine and anesthesiologists in a hybrid operating room.

Forth, further prospective studies are required to confirm these observational results.

## Conclusions

Micra™ VVI and AV LCP implantation in patients requiring an extraction of infected or dysfunctional CIED was feasible, and two-thirds of LCPs could be implanted in a single procedure with CIED extraction. We observed low in-hospital mortality rates with this new therapeutic approach. The causes of death after hospital discharge were not related to LCP implantation or CIED extraction procedures. In our cohort, no recurrent LCP infections were detected during follow-up. Therefore, LCPs appear to be a beneficial therapeutic option for patients who require permanent pacemaker therapy after CIED extraction for infection or dysfunction.

## Abbreviations

AV: Atrioventricular
CIED: Cardiac implantable electronic device
CRT-D: Cardiac resynchronization therapy-defibrillator
CRT-P: Cardiac resynchronization therapy-pacemaker
FDG PET/CT: Fluorodeoxyglucose positron emission tomography/computed tomography
ICD: Implantable cardioverter defibrillator
LCP: Leadless cardiac pacemaker
LV: Left ventricular
RA: Right atrial
RV: Right ventricular
